# RhoA enhances osteosarcoma resistance to MPPa-PDT via the Hippo/YAP signaling pathway

**DOI:** 10.1186/s13578-021-00690-6

**Published:** 2021-10-09

**Authors:** Fangbiao Zhan, Tao He, Zhiyu Chen, Qiang Zuo, Yang Wang, Qiaochu Li, Shenxi Zhong, Yunsheng Ou

**Affiliations:** 1 Department of Orthopedics, The First Affiliated Hospital of Chongqing Medical University, Yuzhong, Chongqing, 400016 China; 2 Department of Orthopedics, Chongqing University Three Gorges Hospital, Wanzhou, Chongqing, 404000 China; 3 Department of Orthopaedic, Chongqing General Hospital, University of Chinese Academy of Sciences, Chongqing, 401147 China

**Keywords:** Osteosarcoma, RhoA, MPPa-PDT, Apoptosis, YAP, Mevalonate

## Abstract

**Background:**

Osteosarcoma (OS) is the most prevalent primary bone malignancy affecting adolescents, yet the emergence of chemoradiotherapeutic resistance has limited efforts to cure affected patients to date. Pyropheophorbide-α methyl ester-mediated photodynamic therapy (MPPa-PDT) is a recently developed, minimally invasive treatment for OS that is similarly constrained by such therapeutic resistance. This study sought to explore the mechanistic basis for RhoA-activated YAP1 (YAP)-mediated resistance in OS.

**Methods:**

The relationship between YAP expression levels and patient prognosis was analyzed, and YAP levels in OS cell lines were quantified. Immunofluorescent staining was used to assess YAP nuclear translocation. OS cell lines (HOS and MG63) in which RhoA and YAP were knocked down or overexpressed were generated using lentiviral vectors. CCK-8 assays were used to examine OS cell viability, while the apoptotic death of these cells was monitored via Hoechst staining, Western blotting, and flow cytometry. Tumor-bearing nude mice were additionally used to assess the relationship between lentivirus-mediated alterations in RhoA expression and MPPa-PDT treatment outcomes. TUNEL and immunohistochemical staining approaches were leveraged to assess apoptotic cell death in tissue samples.

**Results:**

OS patients exhibited higher levels of YAP expression, and these were correlated with a poor prognosis. MPPa-PDT induced apoptosis in OS cells, and such MPPa-PDT-induced apoptosis was enhanced following YAP knockdown whereas it was suppressed by YAP overexpression. RhoA and YAP expression levels were positively correlated in OS patients, and both active and total RhoA protein levels rose in OS cells following MPPa-PDT treatment. When RhoA was knocked down, levels of unphosphorylated YAP and downstream target genes were significantly reduced, while RhoA/ROCK2/LIMK2 pathway phosphorylation was suppressed, whereas RhoA overexpression resulted in the opposite phenotype. MPPa-PDT treatment was linked to an increase in HMGCR protein levels, and the inhibition of RhoA or HMGCR was sufficient to suppress RhoA activity and to decrease the protein levels of YAP and its downstream targets. Mevalonate administration partially reversed these reductions in the expression of YAP and YAP target genes. RhoA knockdown significantly enhanced the apoptotic death of OS cells in vitro and in vivo following MPPa-PDT treatment, whereas RhoA overexpression had the opposite effect.

**Conclusions:**

These results suggest that the mevalonate pathway activates RhoA, which in turn activates YAP and promotes OS cell resistance to MPPa-PDT therapy. Targeting the RhoA/ROCK2/LIMK2/YAP pathway can significantly improve the efficacy of MPPa-PDT treatment for OS.

**Supplementary Information:**

The online version contains supplementary material available at 10.1186/s13578-021-00690-6.

## Background

Osteosarcoma (OS) is the most prevalent form of malignancy affecting adolescents, occurring preferentially in males and developing at relatively high rates in individuals between the ages of 60 and 80 years [1, 2]. While neoadjuvant chemotherapy has led to significant improvements in the duration of OS patient overall and disease-free survival (DFS), the survival of OS patients has plateaued in recent years [1, 3, 4]. The 5-year event-free survival (EFS) of OS patients with lung metastases is just 18.9% [5]. As such, it is critical that the mechanisms governing OS onset and progression be more fully clarified in order to identify novel biomarkers and treatment strategies capable of improving patient outcomes.

Photodynamic therapy (PDT) is a minimally invasive tumor treatment strategy [6, 7]. Patients are initially intravenously administered a photosensitizing agent that accumulates within tumor sites, after which a particular wavelength of visible light is used to irradiate the tumor. The photosensitizer damages and kills tumor cells via a photochemical reaction, aiding in tumor clearance. Appropriate photosensitizer selection is critical in order to maximize PDT efficacy. Pyropheophorbide-a methyl ester (MPPa) is a chlorophyll-derived, second-generation photosensitizer that is stable, readily absorbed, has a defined chemical structure, and exhibits robust photoelectric sensitivity [8–10]. Despite clear evidence that MPPa-PDT can promote the death of tumor cells [8–12], our previous studies showed that the apoptosis rate of OS cells induced by MPPa-PDT treatment was about 40% [8–10], and the therapeutic effect has the potential to be improved. Clarifying the mechanistic basis for such MPPa-PDT resistance is thus important as a means of improving therapeutic outcomes in OS patients.

RhoA is a Rho GTPase that serves as a key regulator of cytoskeletal function, and that also influences the development of chemoresistance [13]. While RhoA activation has been shown to promote certain aspects of tumorigenesis in vitro and in vivo, in some contexts it has also been shown to inhibit particular tumor functions [14, 15]. The relationship between RhoA and MPPa-PDT resistance and the underlying mechanistic association between RhoA and PDT-induced apoptotic cell death remain to be clarified.

The Hippo pathway and its downstream effector Yes-associated protein (YAP), which is a key transcriptional co-activator, can control organ growth and cellular differentiation in the context of development and organ regeneration [16]. Tumor cells generally exhibit YAP hyperactivation, and sustained YAP activity can drive tumorigenesis in mice [16]. YAP can trigger malignant properties including proliferation, chemoresistance, loss of cell polarity, and metastasis [17]. In liver cancer and gastric cancer, YAP has been shown to promote motility and metastasis [18, 19], but there have been few studies to date exploring the mechanistic relationship between RhoA and YAP in the context of PDT-induced OS cell apoptosis.

Recent studies have shown that TYRO3 is activated by extracellular vesicles whereupon it can promote invasive tumor cell metastasis and chemoresistance by activating RhoA or YAP [20]. In gastric cancer, the upregulation of RhoA also enhances tumor invasion and metastasis [21]. The YAP inhibitor verteporfin can reduce tumor recurrence and lung metastasis rates in nude mice [22]. RhoA and YAP have been reported to promote the development of many tumor types, including OS [13, 16, 17, 20–23]. However, the underlying relationship between RhoA and YAP has not been thoroughly studied.

Through a series of experiments, we herein found that human OS tissues exhibit YAP upregulation which is correlated with a poorer prognosis. Increased and reduced YAP functionality respectively suppressed and enhanced OS cell apoptosis, and we further found that the mevalonate pathway was involved in the regulation of RhoA and YAP pathway activity.

## Materials and methods

### OS patient sample analyses

In total, 5 OS patient tumor samples and corresponding paracancerous tissues were collected via biopsy from individuals who had not undergone chemotherapy at the First Affiliated Hospital of Chongqing Medical University. This study was approved by the Ethics Committee of the First Affiliated Hospital of Chongqing Medical University, and all patients provided written informed consent. In addition, results were validated using the Tumor OS public dataset (http://hgserver1.amc.nl/cgi-bin/r2/main.cgi. Accessed 11 April 2020) containing 127 OS samples. Microarray data were analyzed with the R2 platform (http://r2.amc.nl. Accessed 11 April 2020). YAP mRNA and protein levels in different cell lines were assessed using data from the Cancer Cell Line Encyclopedia (CCLE, https://portals.broadinstitute.org/ccle. Accessed 11 April 2020). GraphPad Prism 8 was used to analyze the underlying data.

### Cell culture and treatment

The HOS, MG63, U2OS, and SAOS2 OS cell lines were obtained from the Chinese Academy of Sciences Cell Bank and cultured in DMEM containing 10 % fetal bovine serum (FBS) and penicillin/streptomycin in a humidified 5 % CO_2_ incubator at 37 °C. HOS and MG63 cells were treated with MPPa (Sigma-Aldrich, MO, USA) for 20 h at a range of concentrations while protected from light, after which they were washed twice with PBS. Culture media was then added to appropriate wells, and cells were exposed to red light (630 nm, 40 mW/cm^2^) for 120 s in continuous output mode. For further details regarding this application of MPPa-PDT, refer to our previous study [8].

### Lentivirus transduction

Lentiviral vectors harboring YAP1 and RHOA overexpression vectors (OE-YAP, OE-RHOA) were obtained from Hanbio Biotechnology (Shanghai, China). Amplified primer sequences are listed in Additional file 3: Table S1.

Short hairpin RNA (shRNA) sequences specific for YAP1 (shYAP) or RHOA (shRHOA) were obtained from Hanbio Biotechnology, with target sequences being shown in Additional file 3: Table S2.

HOS and MG63 cells were infected with prepared lentiviral vectors as per provided directions (Additional file 1: Figure S1a, b; Additional file 2: Figure S2a, b), after which qPCR and Western blotting were used to assess changes in target gene expression.

### Reagents

Simvastatin is an inhibitor of HMGCR, which is the rate-limiting enzyme of the mevalonate pathway. Previously, it has been reported that statins exhibit antitumor efficacy [24, 25]. Prior to experimental use, Simvastatin (Selleck, USA, S1792 ) was activated with a solution containing EtOH and NaOH as per provided directions, and was used to treat HOS and MG63 cells at doses of 4 µM and 8 µM respectively (Additional file 1: Figure S1c). The RhoA inhibitor CCG-1423 (Selleck, USA, S7719) and mevalonic acid lithium salt (MedChemExpress, NJ, USA, HY-113,071 A) were used to treat cells at concentrations of 5 µM and 200 mM, respectively (Additional file 1: Figure S1d, e).

### qPCR

TRIzol (TaKaRa, Japan) was used to isolate total RNA from cells, after which the PrimeScript RT Reagent Kit (TaKaRa) was employed to prepare cDNA. A SYBR PrimeScript RT-PCR Kit (TaKaRa) was then used for qPCR based on provided directions with the following thermocycler settings: 3 min at 95 °C; 40 cycles of 95 °C for 15 s, 60 °C for 30 s, and 72 °C for 30 s. GAPDH served as a normalization control. All primers are listed in Additional file 3: Table S3.

### CCK-8 assay

A Cell Counting Kit-8 (CCK-8) assay (Dojindo Molecular Technologies, Kumamoto, Japan) was used to evaluate cell viability. Briefly, cells were added to 96-well plates (5000/well) for 24 h, after which they were treated for 20 h with a range of MPPa concentrations while protected from light. MPPa-PDT was then conducted as detailed previously [8]. Next, 10 µL of CCK-8 solution was added per well and cells were incubated for 1–2 h at 37 °C. Optical density (OD) values were then assessed at 450 nm, with viability being calculated as follows: cell viability (%) = (average OD in the study group-blank)/(average OD in the control group-blank) × 100%.

### Western blotting and RhoA GTPase assay

At 12 h after appropriate treatments, RIPA buffer (Beyotime, Beijing, China) was used to lyse cells, and a BCA kit (Beyotime, China) was utilized to quantify protein concentrations therein. Samples were then boiled, and equal protein amounts from each sample (30 µg) were separated via SDS-PAGE (EpiZyme, China) prior to transfer onto PVDF membranes which were subsequently blocked with 5% BSA or 5% non-fat milk for 2 h at room temperature, and were then incubated overnight at 4 °C with appropriate primary antibodies (Additional file 3: Table S4). Blots were then probed for 1 h with secondary antibodies, and a high sensitivity electrochemiluminescence detection kit was used to detect protein bands with a chemiluminescence imaging system (Bio-Rad, USA). Densitometric analyses of protein bands were conducted with ImageJ.

A Rho Activation Assay Biochem Kit™ (Cat. #BK036) was purchased from Cytoskeleton, Inc. (Acoma St. CO, USA). The GTPase assay was performed according to the provided instructions.

### Immunofluorescence and immunohistochemistry

For immunofluorescence (IF) staining, cells were attached to 24 × 24 mm glass slides, fixed for 30 min with 4% paraformaldehyde at room temperature, and permeabilized for 20 min with 0.5% Triton X-100. Cells were then washed, blocked for 1 h with 5% BSA at room temperature, and incubated overnight at 4 °C with appropriate primary antibodies (Additional file 3: Table S4). After three additional washes with 0.5% PBST (10 min/wash), cells were incubated with secondary antibodies for 1 h while protected from light. Cells were then imaged via fluorescence microscopy. For immunohistochemical (IHC) staining for YAP, cleaved caspase-3, and cleaved PARP, tumor tissue samples were fixed with 4% paraformaldehyde, paraffin-embedded, and cut into serial 4 μm sections using a microtome. Sections were then blocked, stained with appropriate primary antibodies (1:400) (Additional file 3: Table S4), and imaged via microscopy, with the number of cells positive for the proteins of interest being counted in 5 random fields of view at 200× or 400× magnification via light microscopy. Data are reported as the percentage of positive cells.

### Flow cytometry and Hoechst staining

Annexin V/PI or DAPI staining were used to assess cellular apoptosis. Briefly, cells were added to 6-well plates (1 × 10^5^/well) and incubated overnight, after which cells were treated for 20 h with MPPa (0.15 µM for HOS, 0.45µM for MG63), protected from light. At 12 h post-MPPa-PDT, cells were harvested, rinsed with PBS, and stained using Annexin V/PI or DAPI based on provided directions (BD Biosciences, NJ, USA). Frequencies of viable (FITC-/PI- or DAPI-), early apoptotic (FITC+/ PI− or DAPI−), late apoptotic (FITC+/PI + or DAPI+), and necrotic (FITC−/PI + or DAPI+) cells were assessed, with results being presented as the percentage of total apoptotic cells. The cells were counted using a Beckman flow cytometer (CA, USA).

HOS and MG63 cells were added to 24-well plates (5 × 10^4^/well) for 12 h, after which they were washed thrice with PBS, stained for 5 min with 200 µL of Hoechst 33,258 (Beyotime, Beijing, China) at 37 °C protected from light, and cellular apoptosis was then assessed after three more washes via fluorescence microscopy.

### TUNEL staining

Tissue sections were treated with xylene, rehydrated with an ethanol gradient (100% EtOH for 5 min, 100% for 5 min, 85% for 5 min, and 75% for 5 min), treated with 0.5% proteinase K for 20 min at 37 °C, and then treated with Triton X-100 for 20 min. A fluorescent TUNEL Cell Apoptosis Detection Kit (Servicebio, Wuhan, China) was then used based on provided directions. Briefly, TUNEL reaction mixtures (TdT + dUTP mixed at 1:9) were used to stain tissues for 2 h at 37 °C, followed by nuclear staining with DAPI for 8 min at room temperature. A fluorescent microscope was then used to assess apoptosis.

### Xenograft model experiments

The Ethics Committee of the First Affiliated Hospital of Chongqing Medical University approved all animal studies. Nude male BALB/c mice (5 weeks old) obtained from Beijing Huafukang Biotechnology Co. Ltd., HOS cells stably transduced with shNC, shRHOA, or OE-RHOA constructs were subcutaneously implanted into the back of each mouse (1 × 10^6^ cells in 100 µl). All animals were maintained in a specific pathogen-free environment with free food and water access. MPPa-PDT treatment was performed as in prior reports [11]. In total, 30 mice were separated into 6 groups (5/group): shNC, shRHOA, OE-RHOA, shNC + MPPa-PDT, shRHOA + MPPa-PDT group, and OE-RHOA + MPPa-PDT groups. MPPa-PDT treatment was initiated when tumors were 8 ± 1 mm in diameter at a dose of 15 mg/kg MPPa, followed 18 h later by illumination with 120 J/cm^2^ of light at 630 nm every other day for 10 days [11]. Tumors were measured over a 30-day period after the initiation of PDT treatment, with tumor volume (TV) being assessed every third day as follows: TV = 1/2 × a^2^b, where a and b correspond to the short and long axis of the tumor, respectively. On day 30, mice were euthanized, and tumors were collected, weighed, and analyzed. Tumor samples were fixed with 10% formalin, paraffin-embedded, sectioned, and stained with hematoxylin and eosin (H&E) prior to histological imaging via light microscope (200×).

### Statistical analysis

SPSS 26.0 (IL, USA) and GraphPad Prism 8.0 (CA, USA) were used for statistical analyses. Data are means ± standard deviation, and were compared via two-tailed Student’s t test or one-way ANOVAs. Analyses were repeated in triplicate, and P < 0.05 was the significance threshold.

## Results

### OS patients exhibit YAP upregulation that is correlated with a poor prognosis

**Fig. 1:**
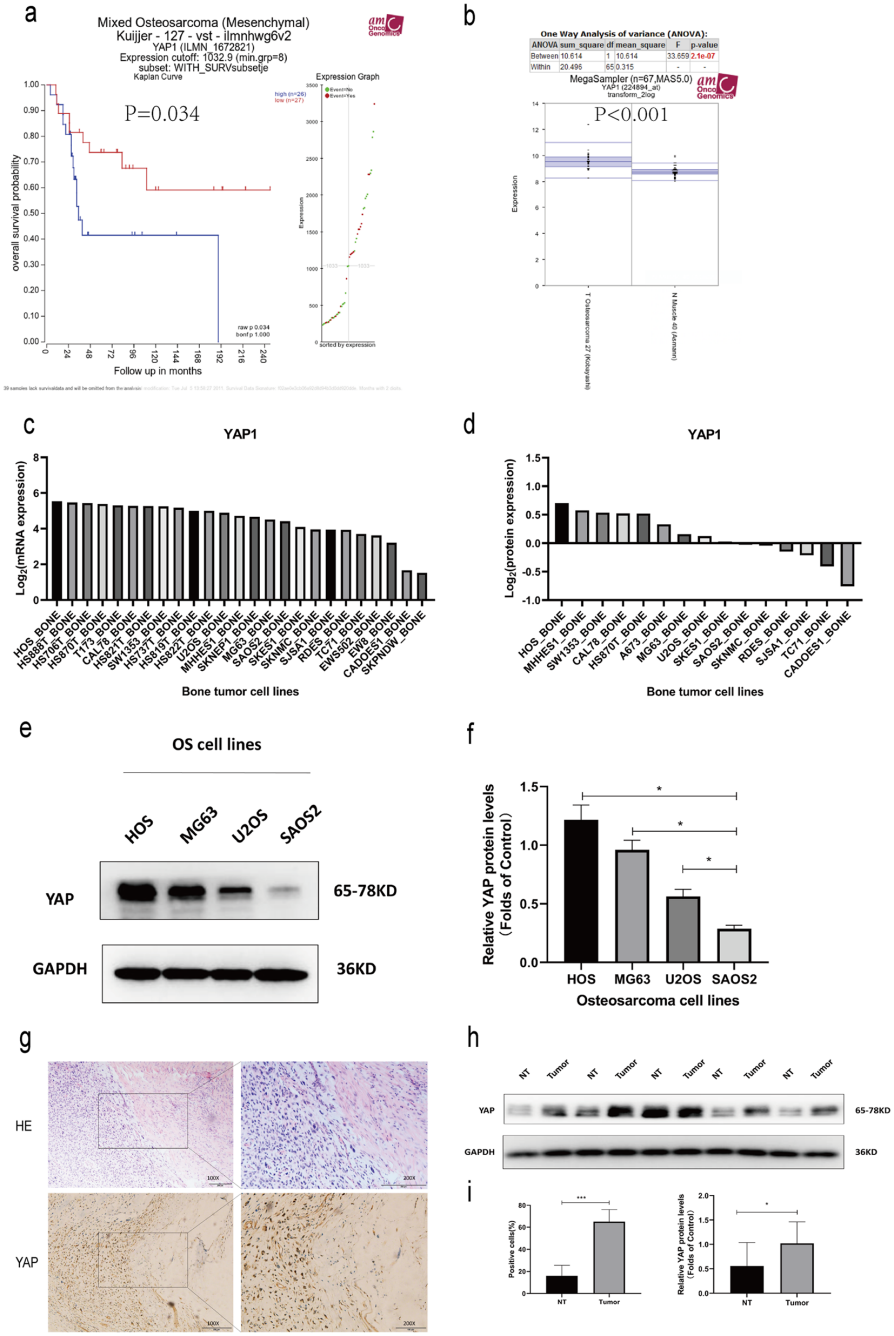
YAP upregulation is associated with a poor OS patient prognosis. **a** Overall survival (OS) outcomes for 26 and 27 patients expressing high and low levels of YAP, respectively. **b** YAP expression levels in 27 OS patient tissue samples and 40 normal patient muscle tissue samples (P < 0.001). **c** Bone tumor cell YAP expression was assessed using Cancer Cell Line Encyclopedia (CCLE) data. **d** YAP protein levels in bone tumor cells were assessed based upon CCLE data. **e**, **f** Western blotting was conducted to assess YAP levels in OS cell lines (HOS, MG63, U2OS, and SAOS2). **g–i** Western blotting and Immunohistochemical staining demonstrating YAP expression levels in human OS tumor cells and paracancerous tissues. Analyses were repeated in triplicate. *P < 0.05 **P < 0.01 ***P < 0.001

To assess YAP expression in OS patients, we began by analyzing mRNA microarray data from the GEO database (GSE42352), revealing that OS patients expressing lower levels of YAP survived for significantly longer on average than did patients expressing higher levels of YAP (Fig. 1a). YAP expression levels were also significantly higher in OS tumor tissues relative to normal muscle (Fig. 1b). When we examined YAP mRNA and protein levels in CCLE data, we found that the HOS cell line exhibited higher levels of YAP expression relative to other bone tumor cell lines (Fig. 1c, d). We then compared these findings to YAP levels in the HOS, MG63, U2OS, and SAOS2 OS cell lines, and similarly confirmed that HOS cells exhibit the highest levels of YAP expression (Fig. 1e, f). We then conducted Western blotting, H&E staining, and IHC staining which confirmed that OS patient tumor tissue samples exhibited higher levels of YAP expression relative to those in paracancerous tissues. These analyses further revealed that YAP was primarily localized to the nucleus of OS tumor cells (Fig. 1g–i). Overall, these results suggested that YAP upregulation may contribute to OS progression.

### MPPa-PDT induces OS cell apoptosis and YAP activation

**Fig. 2:**
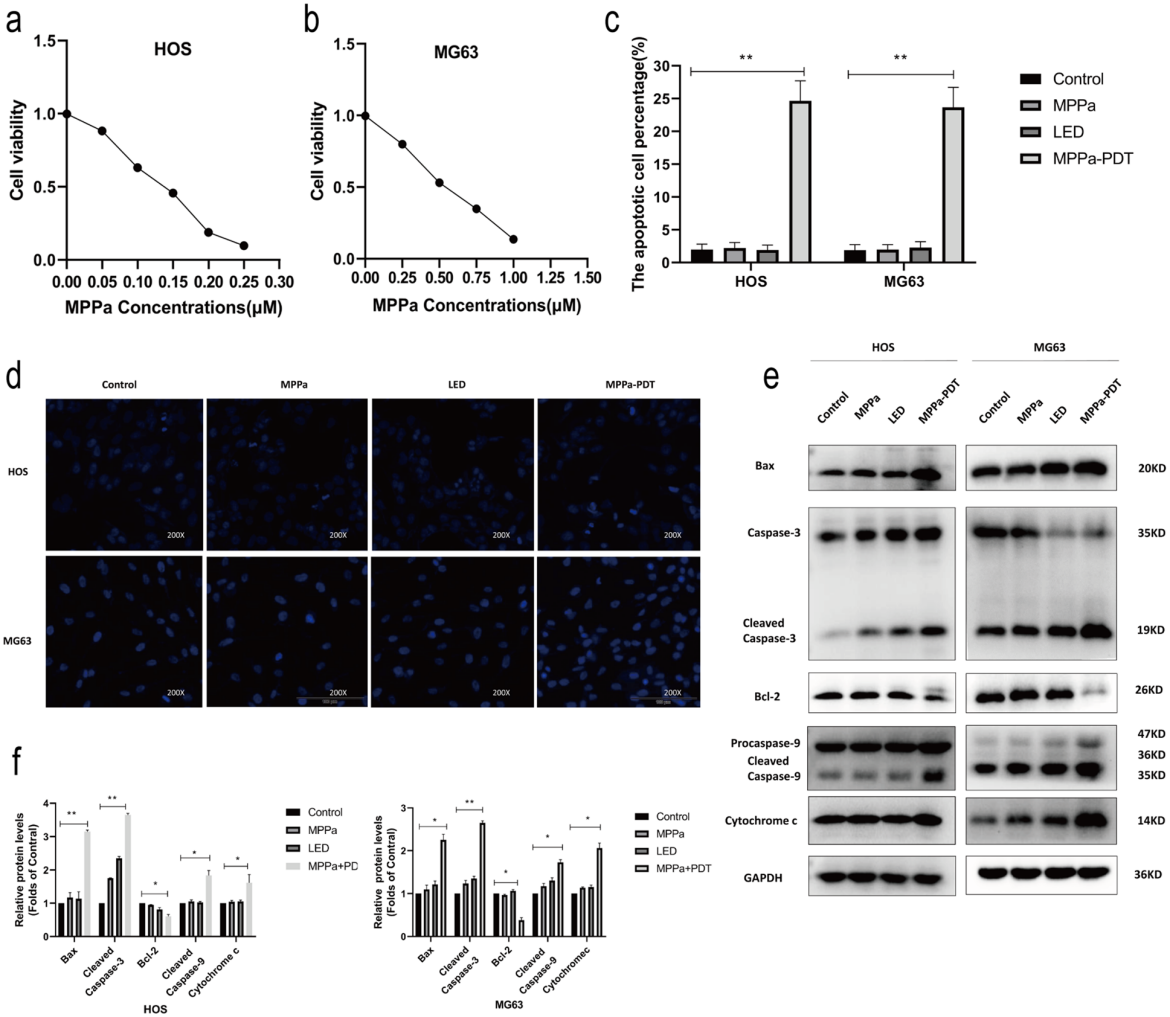

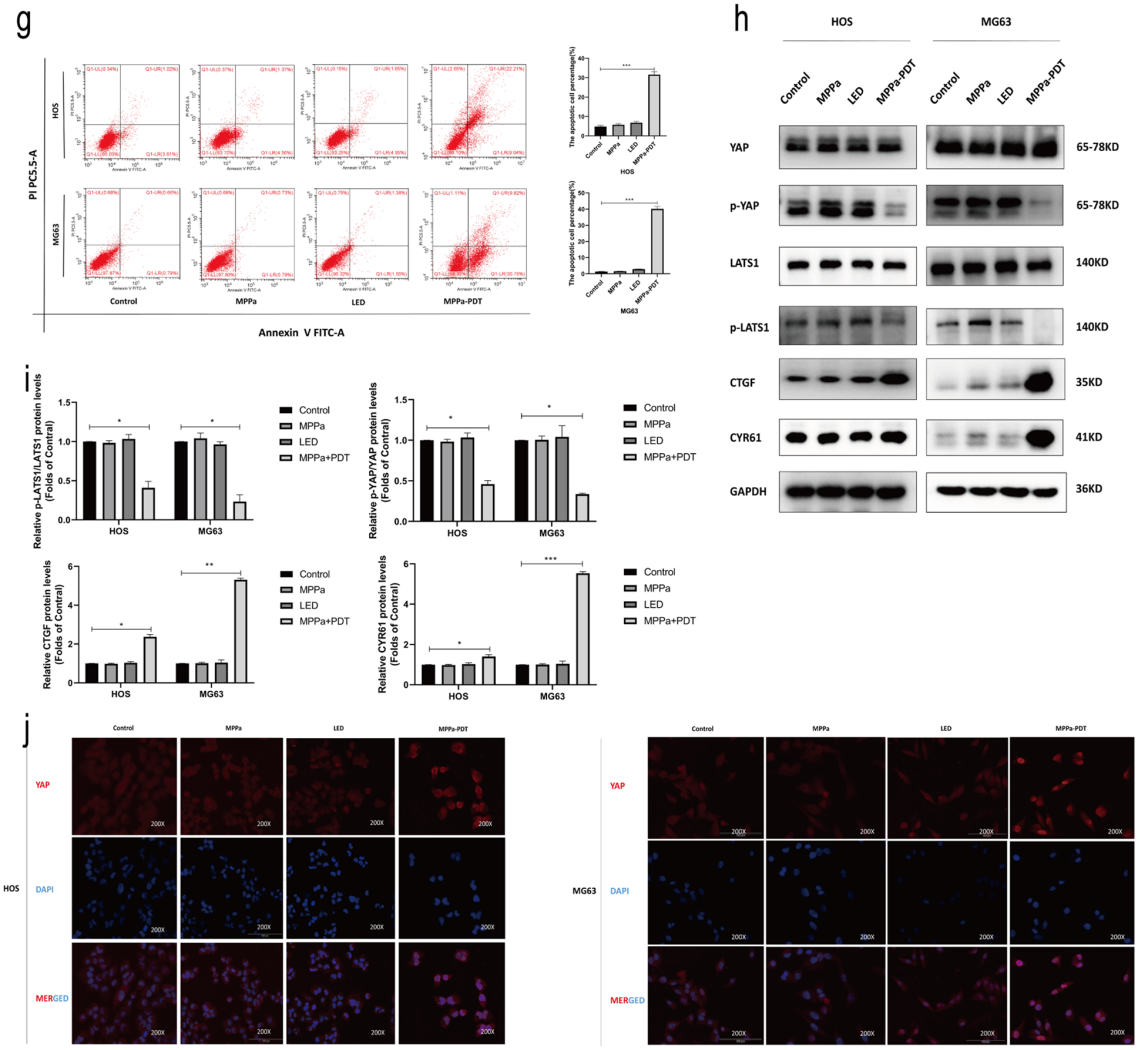
MPPa-PDT induces OS cell apoptosis and YAP activation. **a**,** b** OS cell apoptosis was assessed via CCK-8 assay at 12 h post-MPPa-PDT, revealing MPPa IC50 values of 0.15 µM and 0.45 µM in HOS and MG63 cells, respectively. **c**,** d** Hoechst staining demonstrating the apoptotic death of OS cells at 12 h post-MPPa-PDT. **e**,** f** Western blotting demonstrating apoptosis-related protein expression at 12 h post-MPPa-PDT, with GAPDH as a loading control. **g** HOS and MG63 cell apoptosis rates as assessed via flow cytometry at 12 h post- MPPa-PDT treatment. **h**,** i** Western blotting analyses of protein levels at 12 h post-MPPa-PDT treatment, with GAPDH as a loading control. **j** Immunofluorescence assays demonstrating YAP nuclear translocation within 12 h post-MPPa-PDT. Experiments were repeated in triplicate. * P < 0.05 **P < 0.01 ***P < 0.001

Next, the impact of MPPa-PDT treatment on HOS and MG63 cell viability was assessed in vitro via CCK-8 assay. Following treatment for 24 h, we found that MPPa-PDT induced a dose-dependent reduction in OS cell viability, with MPPa 50% inhibitory concentration (IC50) values of 0.15 µM and 0.45 µM in HOS and MG63 cells, respectively (Fig. 2a, b).

MPPa-PDT-treated cells were then subjected to Hoechst 33,258 staining to assess the induction of apoptotic death via fluorescence microscopy. At 12 h post-MPPa-PDT, we observed significantly increased chromatin density as well as karyopyknosis, condensation, and karyorrhexis, all of which are consistent with apoptotic cell death (Fig. 2c, d). In contrast, reduced apoptotic death was observed in the control group or in cells subjected to MPPa or light exposure in isolation. Western blotting further revealed that there were significant increases in levels of pro-apoptotic proteins (Bax, cleaved caspase-3, cleaved caspase-9, and cytochrome c) and significant reductions in antiapoptotic Bcl-2 levels at 12 h post-MPPa-PDT treatment relative to other treatment groups (Fig. 2e, f).

Annexin V/PI staining was next conducted and cells were evaluated via flow cytometry to quantify the induction of apoptosis. At 12 h post-MPPa-PDT treatment, we observed no significant differences in apoptotic induction among the control, MPPa, and light exposure control groups, whereas significant increases in apoptotic death were observed in the MPPa-PDT group (Fig. 2g). As such, MPPa-PDT can effectively induce the apoptotic death of HOS and MG63 cells.

YAP has previously been linked to chemoresistance in OS [26]. To assess the ability of MPPa-PDT to induce YAP activation in HOS and MG63 cells, we next conducted Western blotting assays which demonstrated reductions in p-LATS/LATS and p-YAP/YAP protein levels at 12 h post-MPPa-PDT relative to the three other treatment groups, together with increases in CTGF and CYR61 protein levels at this same time point (Fig. 2h, i). Immunofluorescent staining confirmed that at 12 h post-MPPa-PDT, YAP was primarily localized to the nucleus, whereas in the three control groups it was instead localized to the cytoplasm (Fig. 2j). Together, these results suggested that MPPa-PDT can induce apoptosis and YAP activation in OS cells.

### YAP enhances OS cell resistance to MPPa-PDT treatment

**Fig. 3:**
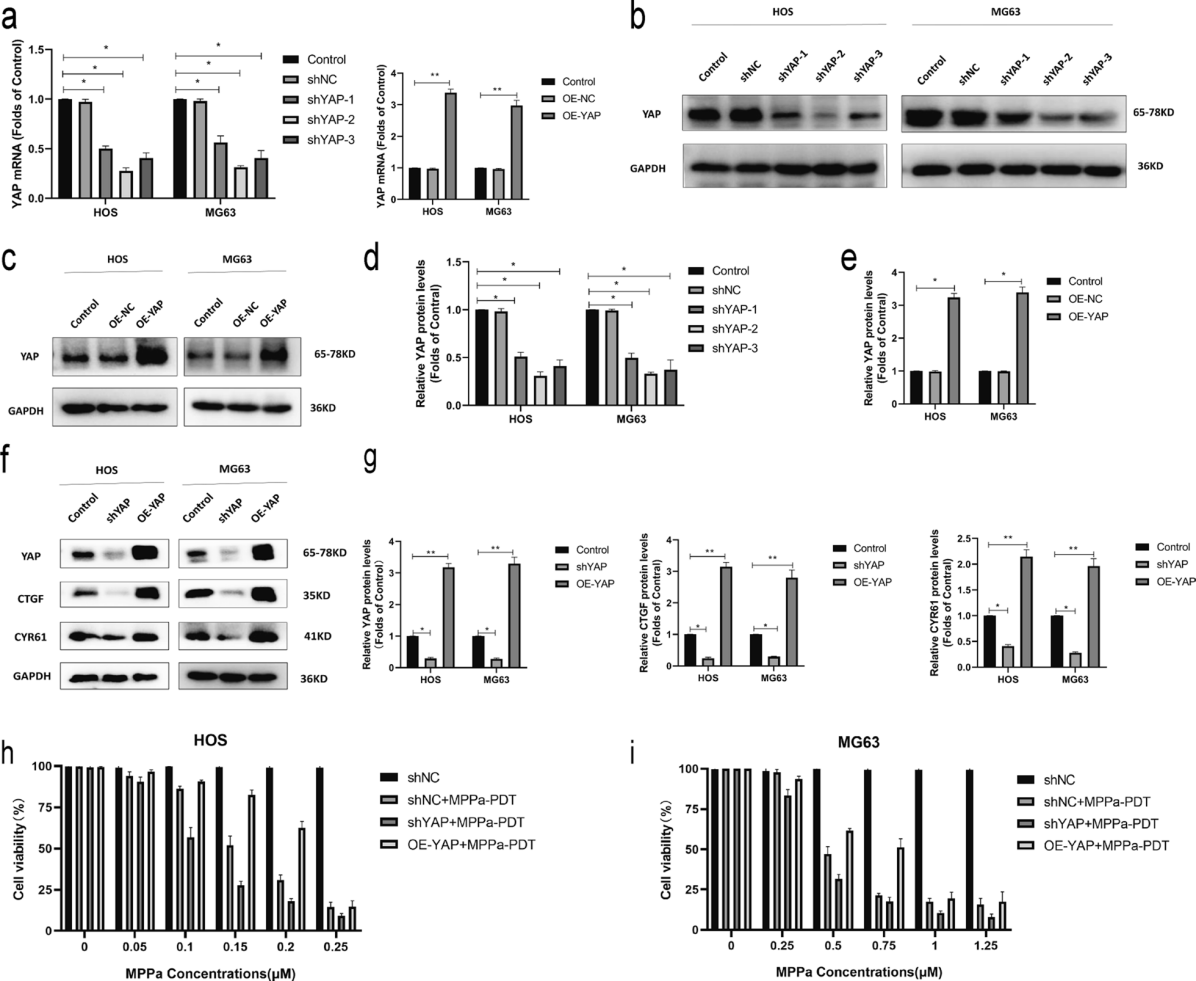

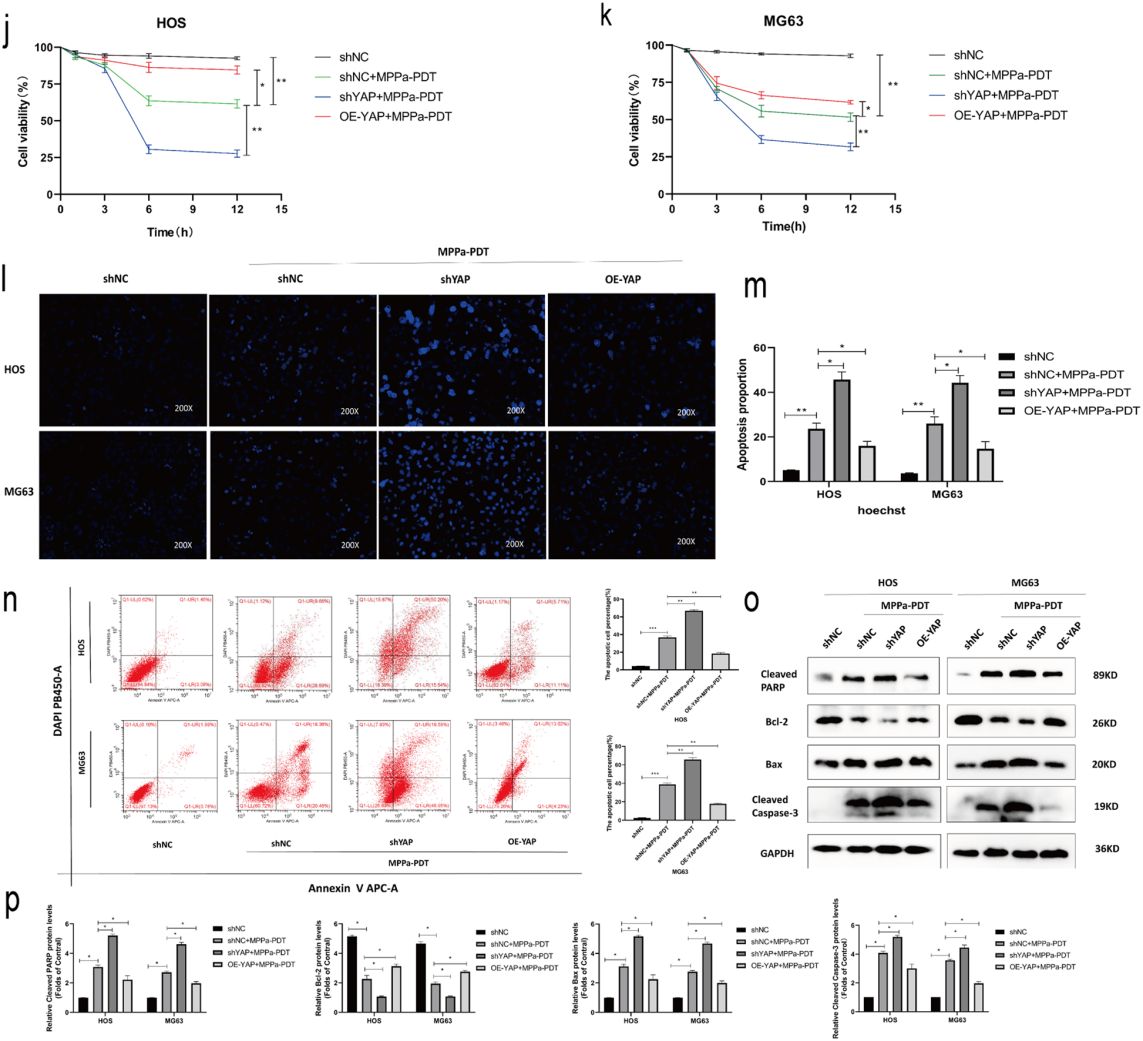
YAP alleviates the MPPa-PDT-induced apoptotic death of OS cells. **a** qPCR was used to assess YAP expression in lentivirally infected HOS and MG63 cells. **b–e** Western blotting demonstrating YAP levels in HOS and MG63 levels in lentivirally infected cells, with GAPDH as a normalization control. **f**,** g** Protein levels of YAP and its downstream targets were assessed via Western blotting, with GAPDH as a normalization control. **h**,** i** Six different human OS cell lines (HOS-shNC, HOS-shYAP, HOS-OE-YAP, MG63-shNC, MG63-shYAP, and MG63-OE-YAP) were treated with various concentrations of MPPa for 20 h and then subjected to PDT, and a CCK-8 assay was used to assess cell viability at 12 h post-MPPa-PDT. **j**,** k** CCK-8 assay results demonstrating changes in OS cell viability following YAP knockdown or overexpression at 12 h post-MPPa-PDT. **l**,** m** Hoechst staining demonstrating the apoptotic death of OS cells at 12 h post-MPPa-PDT following YAP knockdown or overexpression. **n** HOS and MG63 cell apoptosis rates were assessed via flow cytometry at 12 h post- MPPa-PDT treatment. **o**,** p** Western blotting analyses of protein levels at 12 h post-MPPa-PDT treatment, with GAPDH as a loading control. Experiments were repeated in triplicate. * P < 0.05 **P < 0.01 ***P < 0.001

To further establish the functional importance of YAP in OS cells, we next knocked down or overexpressed this gene in HOS and MG63 cells and confirmed these changes in YAP expression via Western blotting and qPCR (Fig. 3a–e). YAP knockdown and overexpression also respectively resulted in significant increases and decreases in the levels of YAP downstream target proteins (CTGF and CYR61) (Fig. 3f, g). CCK-8 assays were used to examine the viability of MPPa-PDT-treated OS cells (HOS and MG63) following YAP knockdown or overexpression. These cells were treated with a wide range of photosensitizer doses in combination with PDT after 12 h. These photosensitizers exhibited a concentration-dependent effect on subsequent PDT outcomes with respect to target cell viability (Fig. 3h, i). At 12 h following MPPa-PDT treatment, the 50% inhibitory concentration (IC50) of MPPa in OS cells was 0.155 µM in HOS-shNC cells, 0.107 µM in HOS-shYAP cells, 1.766 µM in HOS-OE-YAP cells, 0.448 µM in MG63-shNC cells, 0.069 µM in MG63-shYAP cells, and 0.982 µM in MG63-OE-YAP cells, respectively. CCK-8 assays revealed that YAP overexpression was sufficient to prevent MPPa-PDT-induced HOS and MG63 cell apoptosis, whereas YAP knockdown enhanced the ability of MPPa-PDT to drive apoptotic death in these OS cell lines (Fig. 3j, k). Hoechst staining and flow cytometry further confirmed these YAP-dependent changes in OS cell apoptosis (Fig. 3l–n). Similarly, Western blotting indicated that YAP knockdown was associated with increases in MPPa-PDT-induced cleaved PARP, Bax, and cleaved caspase-3 together with decreases in Bcl-2 protein levels, while YAP overexpression had the opposite effect (Fig. 3o, p). Together, these results indicated that YAP can enhance OS cell resistance to MPPa-PDT.

### Mevalonate pathway-induced RhoA activation following MPPa-PDT treatment results in enhanced YAP activity

**Fig. 4:**
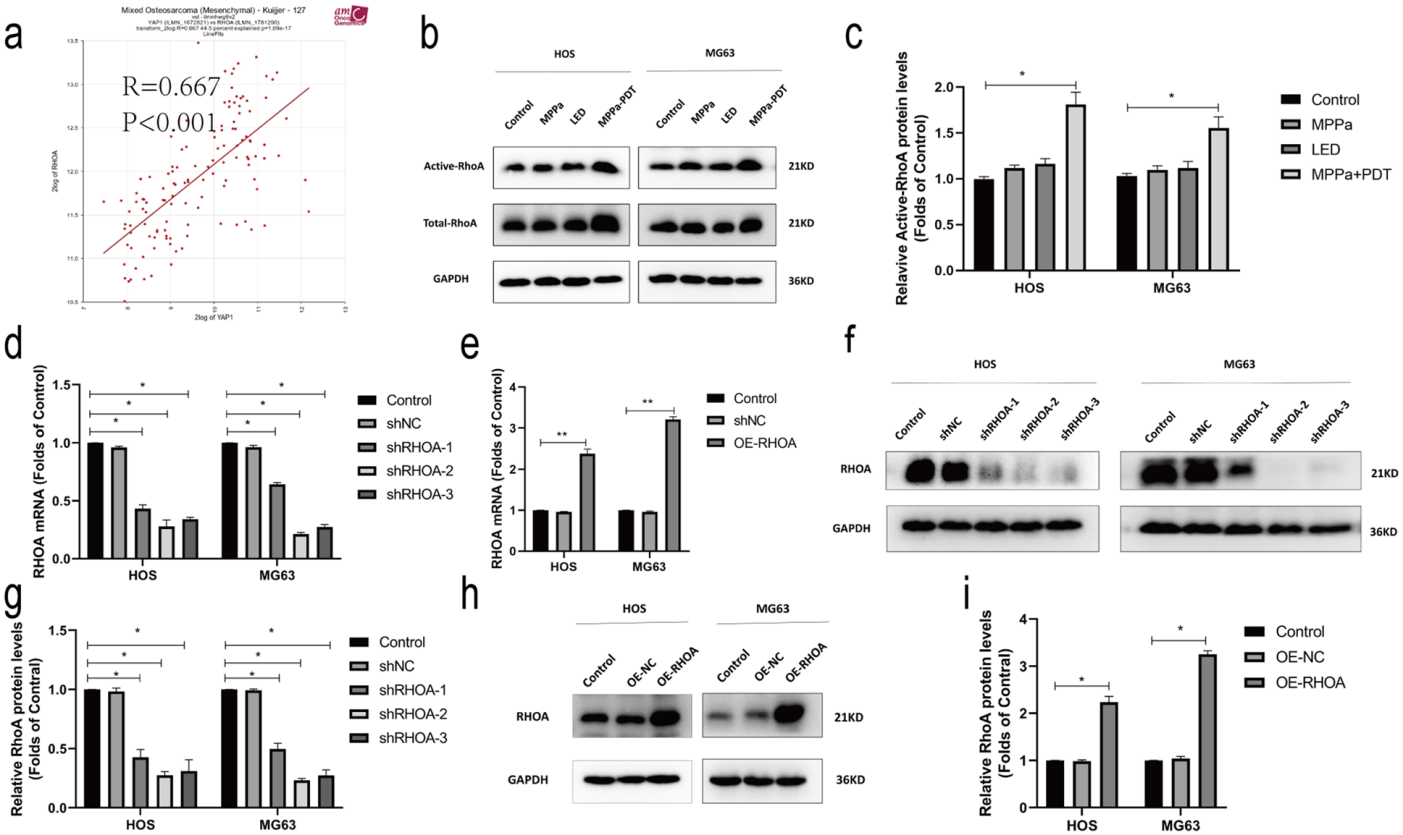

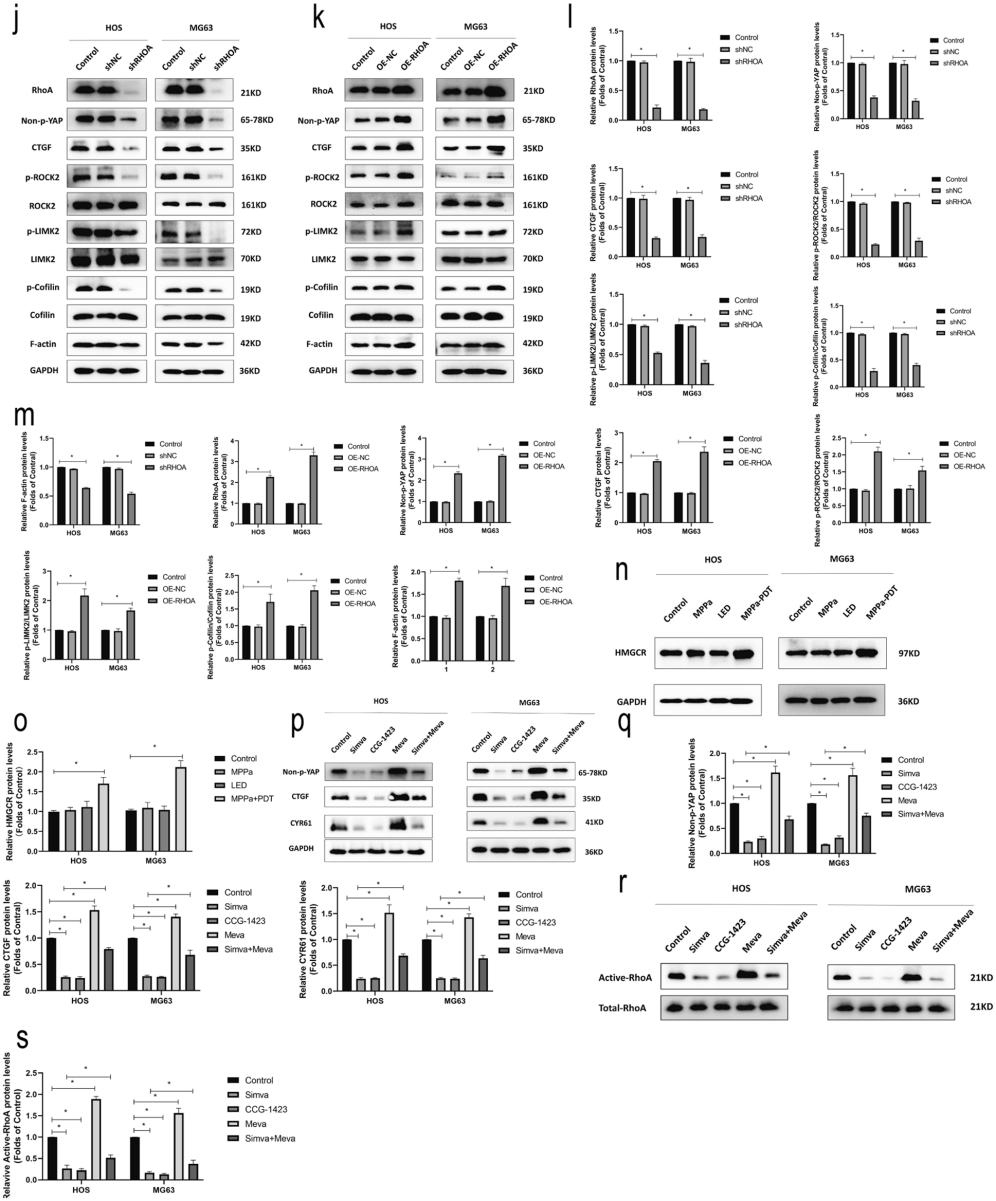
MPPa-PDT activates RhoA via the mevalonate pathway, in turn driving YAP activation. **a** YAP expression and RhoA expression were highly positively correlated in OS samples (r = 0.667, P < 0.001). **b**,** c** Total RhoA and active RhoA (RhoA-GTP) expression in HOS and MG63 cells was assessed by the GTPase assay and western blotting at 12 h post-MPPa-PDT. **d**,** e** RhoA expression was assessed via qPCR in OS cells following RhoA lentivirus infection. **f–i** RhoA protein levels in OS cells following RhoA knockdown or overexpression were assessed via Western blotting. **j–m** Changes in RhoA protein levels following RhoA knockdown or overexpression were assessed via Western blotting. **n**,** o** HMGCR protein levels were assessed via Western blotting in HOS and MG63 cells at 12 h post-MPPa-PDT treatment. **p**,** q** HOS and MG63 cells were treated with mevalonate (200 mM) for 6 h, simvastatin (4 mM for HOS, 8 mM for MG63) for 24 h, or CCG-1423 (5 μm) for 48 h, after which protein expression was assessed via Western blotting. **r**, **s **HOS and MG63 cells were treated with mevalonate (200 mM) for 6 h, simvastatin (4 mM for HOS, 8 mM for MG63) for 24 h, or CCG-1423 (5 μm) for 48 h, after which a GTPase assay revealed changes in levels of active RhoA, with total RhoA serving as a normalization control. Experiments were repeated in triplicate. GAPDH served as a normalization control in all Western blotting assays unless otherwise noted. * P < 0.05 **P < 0.01 ***P < 0.001

Bioinformatics analyses suggested a positive correlation between YAP and RhoA expression in OS samples (Fig. 4a). RhoA-GTPases pull-down assays showed that RhoA activities were significantly upregulated at 12 h post-MPPa-PDT relative to the three other treatment groups (Fig. 4b, c). Western blotting further confirmed that RhoA protein levels were elevated in HOS and MG63 cells at 12 h post-MPPa-PDT relative to the three other treatment groups (Fig. 4b, c). We then used shRNA and lentiviral constructs to knockdown or overexpress RhoA in these OS cell lines, confirming these changes by qPCR and Western blotting (Fig. 4d–i). These changes in RhoA expression were also associated with changes in the expression of RhoA/ROCK2/LIMK2 pathway proteins, activated YAP (S127, unphosphorylated), and the YAP downstream target CTGF (Fig. 4j–m). Recent evidence suggests that chemotherapy can enhance HMGCR protein levels in the context of acute myeloid leukemia (AML) such that statins may represent effective anticancer treatments [27, 28]. We therefore assessed HMGCR protein levels via Western blotting and found that these levels were significantly elevated 12 h following MPPa-PDT relative to other treatment groups (Fig. 4n, o). To additionally evaluate associations between the activation of RhoA and YAP, we then treated OS cells with simvastatin, CCG-1423 (a RhoA inhibitor), mevalonate, and simvastatin + mevalonate, revealing that both simvastatin and CCG-1423 significantly suppressed the activation of YAP and its downstream target genes, whereas mevalonate was able to partially rescue YAP activation (Fig. 4p, q). Through RhoA-GTPases pull-down assays, we found that simvastatin and CCG-1423 significantly inhibited RhoA activation, while mevalonate was able to partially reverse this phenotype (Fig. 4r, s). Together, these data suggested that the mevalonate pathway facilitated MPPa-PDT-induced RhoA activation, in turn driving YAP activity in OS cells.

### RhoA enhances OS cell resistance to MPPa-PDT treatment

**Fig. 5:**
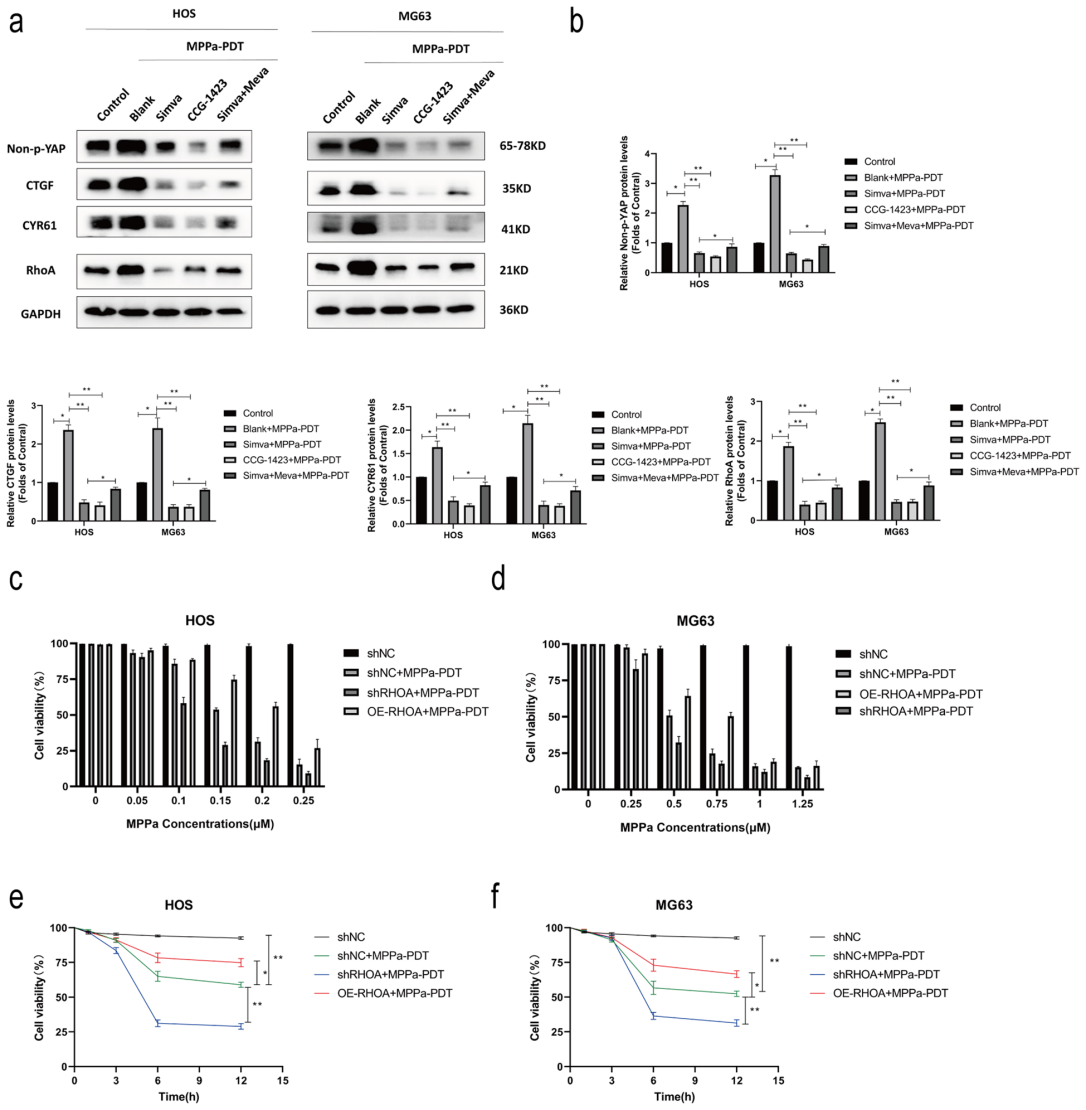

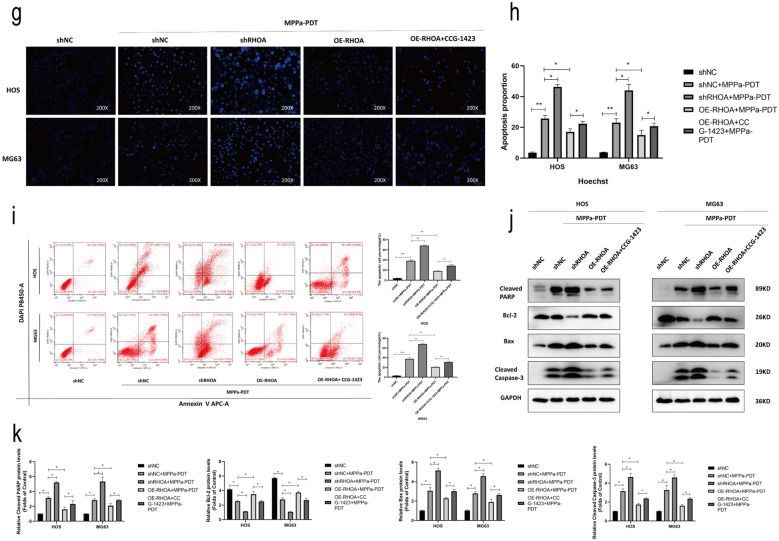
RhoA promotes OS cell resistance to MPPa-PDT. **a**,** b** Prior to MPPa-PDT, HOS and MG63 cells were treated with mevalonate (200 mM) for 6 h, simvastatin (4 mM for HOS, 8 mM for MG63) for 24 h, or CCG-1423 (5 μm) for 48 h, respectively. Western blotting was used to assess protein levels at 12 h post-MPPa-PDT, with GAPDH as a loading control. **c**,** d** Six different human OS cell lines (HOS-shNC, HOS-shRHOA, HOS-OE-RHOA, MG63-shNC, MG63-shRHOA, and MG63-OE-RHOA) were treated with various concentrations of MPPa for 20 h and then subjected to PDT, with a CCK-8 assay being used to assess cell viability at 12 h post-MPPa-PDT treatment. **e**,** f** Changes in OS cell viability following RhoA knockdown or overexpression were assessed at 12 h post-MPPa-PDT. **g**, **h** Hoechst staining demonstrating changes in OS cell apoptosis following RhoA knockdown or overexpression at 12 h post-MPPa-PDT. CCG-1423 treatment (5 μm for 48 h) was able to rescue the inhibition of apoptosis caused by the overexpression of RhoA. **i** HOS and MG63 cell apoptosis rates were assessed via flow cytometry. **j**, **k** Western blotting analyses of protein levels at 12 h post-MPPa-PDT treatment, with GAPDH as a loading control. Experiments were repeated in triplicate. * P < 0.05 **P < 0.01 ***P < 0.001

To further establish the functional importance of RhoA activation and YAP in the context of MPPa-PDT treatment of OS cells, we next conducted Western blotting analyses of the following treatment groups: control, MPPa-PDT, simvastatin + MPPa-PDT, CCG-1423 + MPPa-PDT, and mevalonate + simvastatin + MPPa-PDT groups. This analysis revealed that simvastatin and CCG-1423 significantly reduced the levels of RhoA, activated YAP, and downstream target genes in these cells following MPPa-PDT treatment, while YAP activation was rescued by mevalonate and inhibited by simvastatin (Fig. 5a, b). CCK-8 assays were used to examine the viability of MPPa-PDT-treated OS cells (HOS and MG63) following RHOA knockdown or overexpression. These cells were treated with a wide range of photosensitizers in combination with PDT after 12 h. These photosensitizers exhibited a concentration-dependent effect on subsequent PDT outcomes with respect to target cell viability (Fig. 5c, d). At 12 h following MPPa-PDT treatment, the IC50 of MPPa in OS cells was 0.158 µM in HOS-shNC cells, 0.111 µM in HOS-shRHOA cells, 3.460 µM in HOS-OE-RHOA cells, 0.456 µM in MG63-shNC cells, 0.104 µM in MG63-shRHOA cells, and 0.801 µM in MG63-OE-RHOA cells, respectively. To examine the effects of RhoA in OS cells upon MPPa-PDT treatment, CCK-8 assays were next performed, indicating that RhoA overexpression was sufficient to reverse the apoptotic death of HOS and MG63 cells following such treatment, whereas RhoA knockdown had the opposite effect (Fig. 5e, f). Hoechst staining and flow cytometry further confirmed these results. The application of the RhoA inhibitor CCG-1423 was sufficient to rescue the inhibition of apoptosis caused by RhoA overexpression. (Fig. 5g–i). Western blotting also revealed that RhoA overexpression was able to partially reverse MPPa-PDT-induced apoptosis in HOS and MG63 cells, whereas RhoA knockdown promoted such apoptosis as evidenced by increases in levels of cleaved PARP, Bax, and cleaved caspase-3 together with reductions in Bcl-2 levels (Fig. 5j, k). Together, these data suggested that RhoA can enhance OS cell resistance to MPPa-PDT.

### RhoA knockdown enhances the in vivo sensitivity of OS tumors to MPPa-PDT treatment

**Fig. 6:**
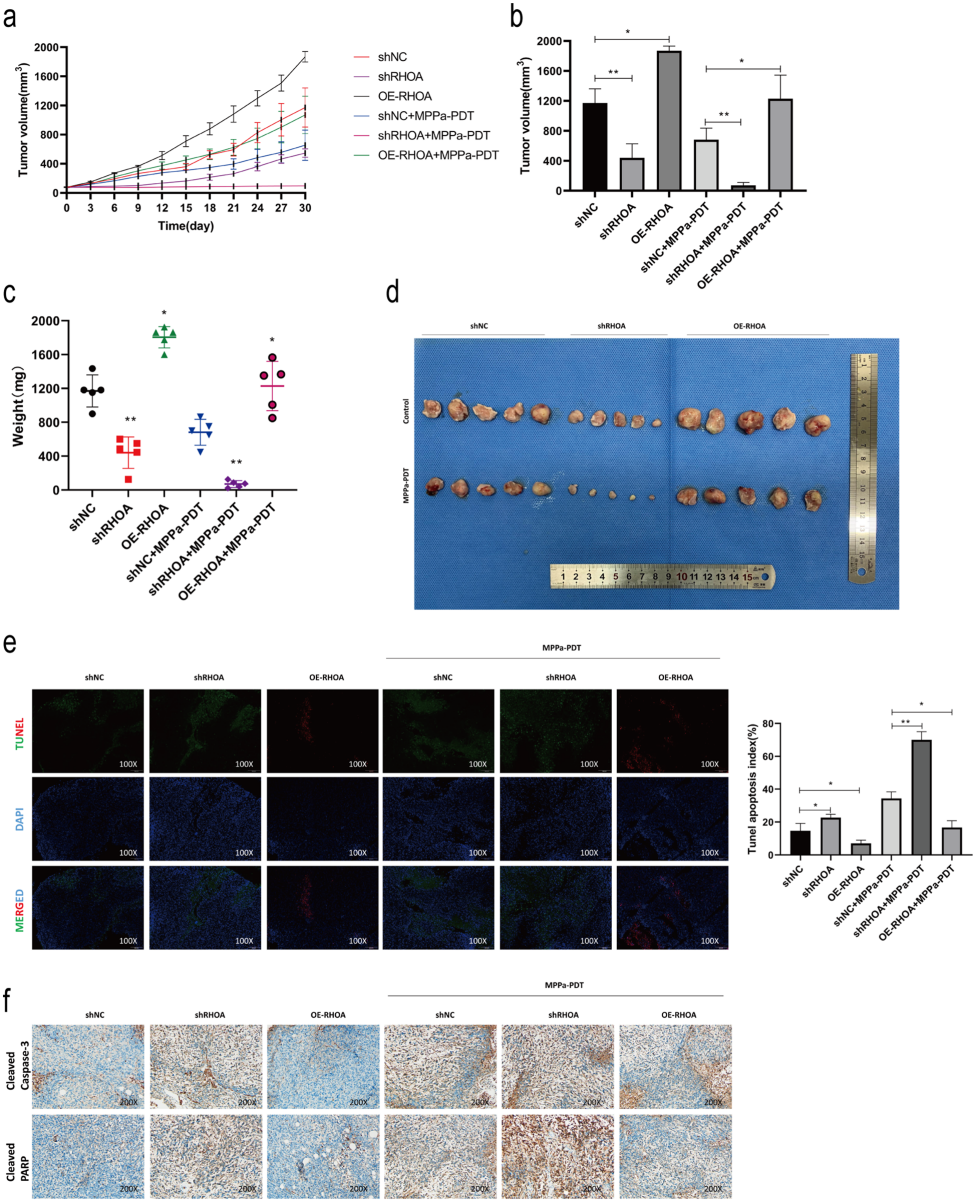
RhoA promotes the in vivo resistance of OS cells to MPPa-PDT. **a** Nude mice were subcutaneously implanted with HOS cells stably transfected with shNC, shRHOA, or OE-RHOA (n = 5/group), and tumor diameters were monitored over time. **b–d** On day 30 post-MPPa-PDT treatment initiation, mice were euthanized and tumor volume and weight values were recorded, after which tumors were imaged. **e** TUNEL staining of tumor tissue sections was performed, and apoptotic index values were calculated. **f** Tumor immunohistochemical staining was performed to assess changes in apoptotic marker expression. * P < 0.05 **P < 0.01 ***P < 0.001

Given that knocking down RhoA inhibited OS cell malignancy in vitro, we next sought to confirm these findings in vivo in a xenograft mouse model system. Mice implanted with shRHOA tumors exhibited significantly reduced tumor growth relative to mice in the shNC group (Fig. 6a–d), whereas OE-RHOA animals exhibited significantly enhanced tumor growth. The greatest inhibition of tumor growth was observed for mice in the shRHOA group that were subjected to MPPa-PDT treatment. In contrast, the MPPa-PDT treatment of mice in the OE-RHOA tumor group was associated with more robust tumor growth as compared to MPPa-PDT treatment in shNC mice. Next, tumor tissue sections were subjected to TUNEL staining in order to evaluate apoptotic cell death following MPPa-PDT treatment, revealing variable apoptotic index values in the different treatment groups (shRHOA, OE-RHOA, MPPa-PDT, shRHOA/MPPa-PDT, and OE-RHOA/MPPa-PDT) (Fig. 6e). We additionally assessed levels of key apoptotic markers including cleaved caspase-3 and cleaved PARP to confirm the ability of RhoA knockdown and MPPa-PDT to suppress OS tumor growth, revealing significant increases in the levels of both of these cleaved proteins in the shRHOA and MPPa-PDT groups relative to the control group. Notably, levels of these apoptotic proteins were highest in the combination shRHOA/MPPa-PDT treatment group relative to these other treatment groups (Fig. 6f). Together, these data suggested that RhoA knockdown alone or in combination with MPPa-PDT was sufficient to inhibit OS tumor growth in vivo, with a combination of RhoA knockdown and MPPa-PDT treatment being associated with optimal outcomes indicating that knocking down this Rho GTPase can abolish MPPa-PDT resistance.

## Discussion

Efforts to effectively treat OS have stagnated in recent years owing to OS tumor resistance to traditional therapeutic approaches [4], emphasizing the need for novel treatment modalities [6]. MPPa-PDT is a minimally invasive antitumor treatment approach that can achieve effective localized benefits without significant side effects. PDT has been used to treat a range of cancer types to date including gastric, lung, breast, bladder, prostate, esophageal, and head and neck tumors [7]. For PDT applications, specific photosensitizer agents can, when exposed to visible light of a particular wavelength, generate large quantities of reactive oxygen species (ROS) that directly induce tumor cell death [8]. We have previously shown that MPPa-PDT treatment was sufficient to induce OS cell apoptosis while impairing their migration and metastatic progression [8, 9], although therapeutic resistance to MPPa-PDT can emerge in these cells [10]. As such, we herein sought to explore the mechanistic basis for MPPa-PDT resistance in OS cells. In line with our previous data, we determined that MPPa-PDT was able to induce apoptotic cell death in HOS and MG63 cells [8, 9]. In one recent study, MPPa-PDT was shown to suppress the proliferation of breast cancer cells via ER stress-induced autophagy [11]. Suzuki et al. [7] have also reported that MPPa-PDT can interfere with tumor vascularization. We found that OS cells and patient tumor samples exhibited high levels of YAP expression, and that MPPa-PDT was sufficient to induce OS cell apoptotic death and YAP activation, highlighting a potential mechanism whereby MPPa-PDT treatment resistance may emerge in this oncogenic context.

**Fig. 7:**
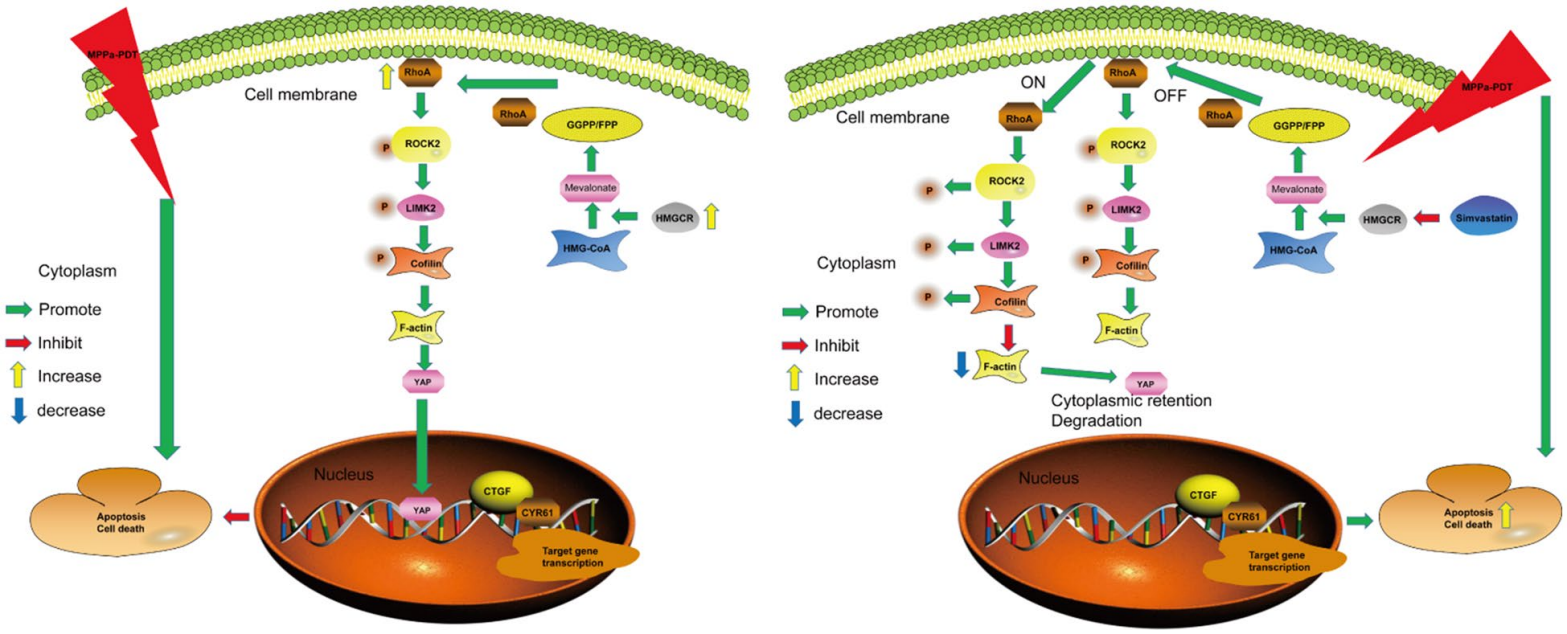
The potential mechanisms of RhoA-mediated MPPa-PDT resistance in OS. The mevalonate pathway activates RhoA, in turn leading to YAP activation and MPPa-PDT resistance in OS cells

YAP is an effector molecule within the Hippo signaling pathway that is an important regulator of tissue and organ development [16]. We observed a strong positive correlation between RhoA and YAP gene expression in OS. Aberrant RhoA activation can, in turn, induce YAP activation leading to proliferation, metastasis, and epithelial-mesenchymal transition in endometrial cancer cells [29]. Vigneswaran et al. found that the YAP inhibitor verteporfin was able to enhance therapeutic sensitivity in EGFR mutant glioblastoma multiforme cells [30]. We similarly determined that OS patients exhibited high levels of YAP expression, with increased YAP expression being associated with decreases in patient overall survival and a worse overall prognosis. YAP has been repeatedly shown to promote tumor cell resistance to radiotherapy, chemotherapy, and targeted treatment [17, 31]. We detected YAP activation in OS cells following MPPa-PDT treatment, in line with prior evidence demonstrating YAP activation following chemoradiotherapeutic treatment [26, 32]. The knockdown of YAP was sufficient to enhance OS cell MPPa-PDT sensitivity in vitro, whereas YAP overexpression had the opposite effect. As an oncoprotein, YAP can suppress tumor cell apoptosis while promoting proliferative activity. YAP is subject to regulation by several upstream proteins in the Hippo signaling pathway, including LATS1/2 [16]. Zucchini et al. [33] determined that ROCK2 can enhance YAP activation, thereby promoting OS cell proliferation and metastasis. Tocci et al. [34] reported that the RhoA/actin-dependent pathway can regulate YAP-mediated chemoresistance in ovarian cancer. Following RhoA knockdown, ROCK2 and LIMK2 phosphorylation levels decline, decreasing F-actin expression and thereby decreasing levels of unphosphorylated YAP and its downstream target genes. We found that YAP activity levels in OS were controlled by RhoA through the RhoA/ROCK2/LIMK2 signaling axis (Fig. 7a, b). YAP expression levels were strongly positively correlated with those of RhoA, indicating that RhoA regulates YAP activity in OS.

Several recent studies have explored the functional importance of RhoA and other Ras GTPases [15, 23, 29, 35, 36], with RhoA hyperactivation having been linked to proliferation and chemoresistance in gastric, bladder, and non-small cell lung cancer [15, 20, 37, 38]. The importance of RhoA-mediated YAP modulation following MPPa-PDT treatment in OS, however, has not been previously studied. Herein, we found that RhoA was upregulated in OS cells following MPPa-PDT treatment, and that RhoA expression levels were highly correlated with YAP levels. We thus hypothesized that RhoA may serve as a mediator of MPPa-PDT resistance in OS cells through its ability to induce YAP activation. When RhoA was knocked down, we found that OS cells were more sensitive to MPPa-PDT, whereas RhoA overexpression had the opposite effect. HMGCR was recently reported to play a role in chemoresistance in the context of AML [27]. We thus next measured HMGCR expression levels in OS cells and found that it was upregulated following MPPa-PDT treatment. The mevalonate pathway is a key regulator of RhoA activation [25, 39]. Following mevalonate pathway or RhoA inhibition, we detected the inhibition of YAP activation and downstream target gene expression. Together, these data suggested that MPPa-PDT treatment induced mevalonate pathway activation in OS cells, in turn driving YAP activation and enhancing cell survival. Ren et al. [35] reported that endogenous PDL-2 signaling can drive the migratory and metastatic activity of OS cells via the RhoA and autophagy pathways. Park et al. [20] determined that circulating vessels are able to activate RhoA in NSCLC, in turn mediating tumor cell proliferation, migration, and chemoresistance. Hu et al. [37] found that the long noncoding RNA RNA ktn1-as1 was able to promote RhoA activation, thereby driving bladder cancer cell proliferation and consequent tumor progression. These findings thus suggest that RhoA activation is a key determinant of cancer progression.

HMGCR is a rate-limiting enzyme that regulates cholesterol synthesis. Farnesyl pyrophosphate (FPP) and geranylgeranyl pyrophosphate (GGPP) are intermediate products of cholesterol synthesis via the mevalonate pathway. These lipid metabolites act on the post-translational modification of proteins in a range of oncogenic contexts [40]. The membrane localization and activation of RhoA require the assistance of GGPP/FPP [24, 41]. The farnesylation of Ras GTPase, which is regulated by the mevalonate pathway, plays an important role in Ras activation and tumor progression [40–42]. Blocking this mevalonate pathway can inhibit proliferation and induce the apoptotic death of breast cancer cells, while supplementing GGPP can rescue such breast cancer cell apoptosis [25]. In this study, we found that the expression of HMGCR was significantly increased following the MPPa-PDT treatment of OS cells. Subsequently, we used Simvastatin to inhibit the mevalonate pathway, whereupon the expression of activated RhoA was decreased, and the expression of nonphosphorylated YAP (activated YAP) and its downstream target proteins were decreased, indicating that the resistance of OS cells to MPPa-PDT may be driven by the activation of HMGCR. Furthermore, RhoA was activated and YAP activity was increased by the ROCK2/LIMK2/Cofilin pathway, thereby rendering OS cells resistant to MPPa-PDT.

To confirm the role of RhoA as a mediator of the effects of MPPa-PDT treatment in OS cells in vivo, we next utilized mice harboring HOS tumors in which RhoA had been knocked down or overexpressed. RhoA knockdown was associated with significantly reduced tumor growth and increased sensitivity to MPPa-PDT, while RhoA overexpression had the opposite effect. These data suggested that RhoA was able to drive proliferation and to reduce apoptosis in OS cells. As knocking down RhoA was able to suppress tumor growth, a combination of such knockdown and MPPa-PDT treatment may represent a viable synergistic antitumor strategy. Overall, these data suggest that RhoA can enhance OS cell resistance to MPPa-PDT via inducing YAP activation, highlighting RhoA as a promising target for the treatment of OS.

While our human tissue analysis was limited by its relatively small sample size, the selected patients had not undergone chemotherapy treatment prior to biopsy, and the samples are thus representative. We did not assess specific correlations between YAP expression and OS patient clinical stage or prognosis for these patients, but when we assessed previously published datasets we found high YAP expression to correlate with poorer patient prognosis. While YAP knockdown and overexpression were not conducted in vivo, we did knock down and overexpress RhoA in mouse model experiments in the context of MPPa-PDT treatment, demonstrating the key role of RhoA as a promoter of OS cell resistance to MPPa-PDT.

## Conclusions

In summary, we herein found that high levels of YAP expression in OS patients were associated with a poor prognosis. Additionally, the mevalonate pathway was able to regulate RhoA, thereby promoting YAP activation and OS cell resistance to MPPa-PDT treatment. Together, Targeting the RhoA/ROCK2/LIMK2/YAP pathway can significantly improve the efficacy of MPPa-PDT treatment for OS.

## Supplementary Information

**Additional file 1: Figure S1.** Fluorescence images of YAP knockdown or overexpression in osteosarcoma cells treated with the indicated concentrations of simvastatin, CCG-1423, and mevalonate. **a**, **b** The images of osteosarcoma cells were observed under bright field and fluorescence microscopy following the knockdown or overexpression of YAP. **c** A CCK-8 assay was used to assess the viability of HOS and MG63 cells following treatment with different concentrations of simvastatin for 24 h. **d** A CCK-8 assay was used to assess the viability of HOS and MG63 cells following treatment with different concentrations of CCG-1423 for 48 h. **e** Western blotting was used to assess changes in the expression of unphosphorylated YAP in HOS and MG63 cells following a 6 h mevalonate treatment.

**Additional file 2: Figure S2.** Fluorescence images of RHOA knockdown and overexpression in osteosarcoma cells. **a**, **b** Osteosarcoma cells were imaged under bright field and fluorescence microscopy after the knockdown or overexpression of RHOA.

**Additional file 3: Table S1.** Primer sequences used in overexpression lentivirus preparation. **Table S2. ** shRNA target sequences used for knockdown. **Table S3. ** Primer sequences used for PCR amplification. **Table S4. ** The primary antibody used in this experiment and its source.

## Data Availability

The datasets used in this study are available from the corresponding author on reasonable request.
