# Supplementary figures and images for: RhoA enhances osteosarcoma resistance to MPPa-PDT via the Hippo/YAP signaling pathway
Source: Cell Biosci. 2021 Oct 9;11:179. doi: 10.1186/s13578-021-00690-6 (PMC8501741; doi:10.1186/s13578-021-00690-6)

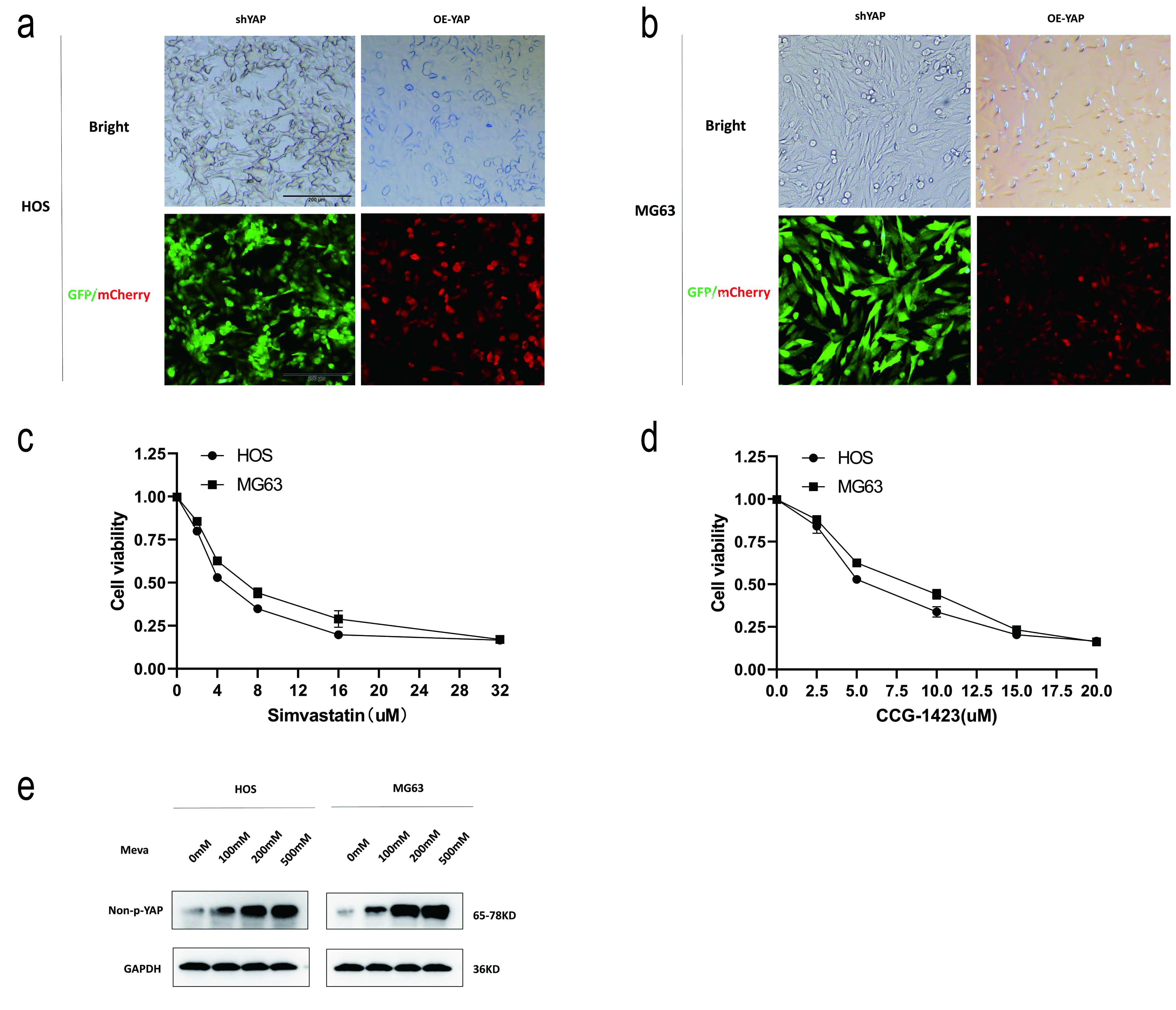

Supplement: Supplementary file 1 — Additional file 1: Figure S1. Fluorescence images of YAP knockdown or overexpression in osteosarcoma cells treated with the indicated concentrations of simvastatin, CCG-1423, and mevalonate. a, b The images of osteosarcoma cells were observed under bright field and fluorescence microscopy following the knockdown or overexpression of YAP. c A CCK-8 assay was used to assess the viability of HOS and MG63 cells following treatment with different concentrations of simvastatin for 24 h. d A CCK-8 assay was used to assess the viability of HOS and MG63 cells following treatment with different concentrations of CCG-1423 for 48 h. e Western blotting was used to assess changes in the expression of unphosphorylated YAP in HOS and MG63 cells following a 6 h mevalonate treatment. [file 13578_2021_690_MOESM1_ESM.tif]

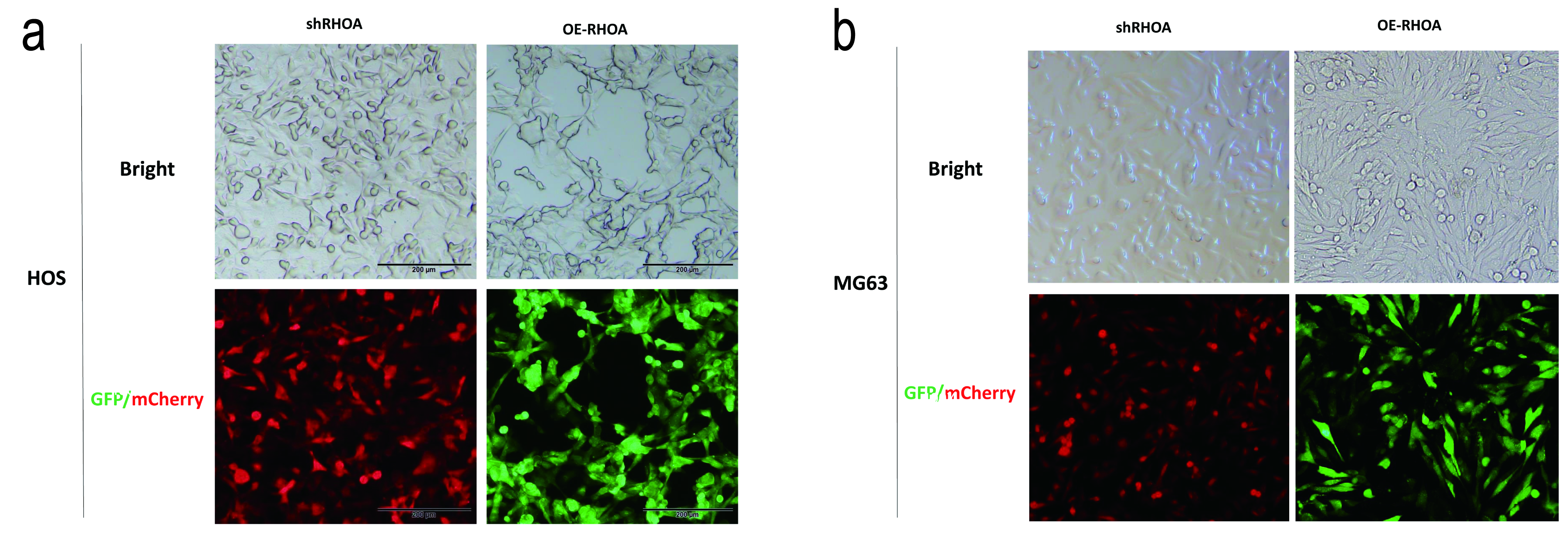

Supplement: Supplementary file 2 — Additional file 2: Figure S2. Fluorescence images of RHOA knockdown and overexpression in osteosarcoma cells. a, b Osteosarcoma cells were imaged under bright field and fluorescence microscopy after the knockdown or overexpression of RHOA. [file 13578_2021_690_MOESM2_ESM.tif]
