# Supplementary material for: RhoA enhances osteosarcoma resistance to MPPa-PDT via the Hippo/YAP signaling pathway
Source: Cell Biosci. 2021 Oct 9;11:179. doi: 10.1186/s13578-021-00690-6 (PMC8501741; doi:10.1186/s13578-021-00690-6)
Supplement: Supplementary file 3 — Additional file 3: Table S1. Primer sequences used in overexpression lentivirus preparation. Table S2. shRNA target sequences used for knockdown. Table S3. Primer sequences used for PCR amplification. Table S4. The primary antibody used in this experiment and its source. [file 13578_2021_690_MOESM3_ESM.docx]

**Table S1. Primer sequences used in overexpression lentivirus.**

| **Gene** | **Primer sequences (5’ to 3’)** |
| --- | --- |
| **YAP1-Forward** | **CAAGTTTGTACAAAAAAGCAGGCT** |
| **YAP1-Reverse** | **ACCCAGCTTTCTTGT ACAAAGTGG** |
| **RHOA-Forward** | **GGTGAATTCGCCACCATGGCTGCCATCCGGAAG** |
| **RHOA-Reverse** | **TGGTACCGAGGATCCCAAGACAAGGCACCCAGA** |

**Table S2. shRNA target sequences used for knockdown.**

| **Gene** | **Target sequence (5’ to 3’)** |
| --- | --- |
| **shNC** | **TTCTCCGAACGTGTCACGTAA** |
| **shYAP1-1** | **CCCGGGATGTCTCAGGAATTGAGAA** |
| **shYAP1-2**  **shYAP1-3** | **GGACTAAGCATGAGCAGCTACAGTG**  **CCTGGGACAAATGTGGACCTTGGAA** |
| **shNC** | **TTCTCCGAACGTGTCACGTAA** |
| **shRHOA-1** | **TGGAAAGACATGCTTGCTCAT** |
| **shRHOA-2** | **GAAAGCAGGTAGAGTTGGCTT** |
| **shRHOA-3** | **GTACATGGAGTGTTCAGCAAA** |

**Table S3. Primer sequences used for PCR amplification**

| **Gene** | **Sequences or Target Sequences** **（5’-3’）** |
| --- | --- |
| **YAP1 (Forward Sequence)**  **YAP1 (Reverse Sequence)**  **RHOA (Forward Sequence)**  **RHOA (Reverse Sequence)**  **GAPDH (Forward Sequence)**  **GAPDH (Reverse Sequence)** | **TGTCCCAGATGAACGTCACAGC**  **TGGTGGCTGTTTCACTGGAGCA**  **TCTGTCCCAACGTGCCCATCAT**  **CTGCCTTCTTCAGGTTTCACCG**  **GTCTCCTCTGACTTCAACAGCG**  **ACCACCCTGTTGCTGTAGCCAA** |

**Table S4. The primary antibody used in this experiment and its source**

| **Primery Antibody** | **Supplier** | **Catalog number** |
| --- | --- | --- |
| **Bax**  **Rabbit mAb** | **Cell signaling Technology (CST)** | **#5023** |
| **Cleaved Caspase-3 Rabbit mAb** | **Cell signaling Technology (CST)** | **#9664** |
| **Bcl-2**  **Rabbit mAb** | **Cell signaling Technology (CST)** | **#4223** |
| **Caspase-9**  **Mouse mAb** | **Cell signaling Technology (CST)** | **#9508** |
| **Cytochrome c**  **Rabbit mAb** | **Cell signaling Technology (CST)** | **#4280** |
| **GAPDH**  **Mouse mAb** | **Proteintech** | **1E6D9** |
| **YAP**  **Rabbit mAb** | **Cell signaling Technology (CST)** | **#14074** |
| **Phospho-YAP (Ser127) Rabbit mAb** | **Cell signaling Technology (CST)** | **#13008** |
| **LATS1**  **Rabbit mAb** | **Cell signaling Technology (CST)** | **#3477** |
| **Phospho-LATS1 (Thr1079) Rabbit mAb** | **Cell signaling Technology (CST)** | **#8654** |
| **CTGF**  **Rabbit mAb** | **Cell signaling Technology (CST)** | **#86641** |
| **CYR61**  **Rabbit mAb** | **Cell signaling Technology (CST)** | **#14479** |
| **Cleaved PARP**  **Rabbit mAb** | **Cell signaling Technology (CST)** | **#5625** |
| **Non-phospho (Active) YAP (Ser127) Rabbit mAb** | **Cell signaling Technology (CST)** | **#29495** |
| ROCK2 Rabbit mAb | **Abcam** | **ab125025** |
| ROCK2 (phospho S1366)Rabbit polyclonal | **Abcam** | **ab228008** |
| LIMK2Rabbit mAb | **Cell signaling Technology (CST)** | #3845 |
| Phospho-LIMK1 (Thr508)/LIMK2 (Thr505) | **Cell signaling Technology (CST)** | #3841 |
| CofilinRabbit mAb | **Cell signaling Technology (CST)** | #5175 |
| Phospho-Cofilin (Ser3) Rabbit mAb | **Cell signaling Technology (CST)** | #3313 |
| F-actin Mouse monoclonal | **Abcam** | ab130935 |
| HMGCR Rabbit monoclonal | **Abcam** | ab174830 |
| RhoA Rabbit mAb | **Cell signaling Technology (CST)** | #2117 |
